# Medical Education in Scotland

**Published:** 1897-09-04

**Authors:** 


### Medical Education in Scotland.

The attractions of Scotland for the medical student are that living is comparatively cheap, and that a sound medical education is crowned with a university degree. These things have drawn students from all parts of the country—indeed, from all parts of the world—to the northern universities. On the benches at Aberdeen, the most northerly of all, one might see the young Scotsman busy "takin' notes" with a "Cockney" on one side of him and a dusky Indian on the other. With good material to work upon, these Scottish schools have produced excellent results. The system, different in certain respects from that of England, has yielded a type of medical man of its own. It is not by his accent alone that one can tell the Scottish doctor; there is an indefinable something in his way of dealing with the problems of disease that speaks of a northern training. It is said that Scottish doctors are popular in England. It may be so; in many cases it is so; but the matter is one as to which one may well hesitate to generalise.

The strong point of the Scottish schools is systematic teaching. The lecture is very much in evidence—too much so, perhaps. It is well, however, that one should be well grounded in principles, always provided that words are not allowed to take the place of things. But it cannot be said that the importance of the laboratory, the museum, and the hospital ward are not realised in Scotland. Resolutely—in some cases handicapped for want of sufficient funds—the Scottish universities have kept abreast of the times in respect of these things.

Edinburgh is the medical as well as the political capital of Scotland. The Edinburgh school has a glorious history, and if it is not so pre-eminent now as it was in the days of Syme and Simpson, may not this be due, in part at any rate, to the coming up of other schools into line? At any rate, with its university, its school of medicine of the Royal Colleges, and its magnificent infirmary, the position of Edinburgh as a centre of medical education is amply assured.

Glasgow holds in education a place worthy of its size and its reputation in the commercial world. In addition to the University there are two extra mural schools —Anderson's College and the Western Medical School. Clinical instruction is provided at the two great hospitals of the city, the Royal and the Western Infirmary.

The University of St. Andrew's and University College, Dundee, are incorporated. Some of the science classes may be taken at St. Andrew's, one of the most attractive residential towns in Scotland. The Royal Infirmary at Dundee, where the clinical teaching is given, has 286 beds.

Some years ago the Royal Infirmary at Aberdeen was enlarged, and more recently the medical school buildings at Marischal College, and Aberdeen is now, in the matter of buildings, one of the best equipped schools in the country.

